# Cross-Regulation of Protein Stability by p53 and Nuclear Receptor SHP

**DOI:** 10.1371/journal.pone.0039789

**Published:** 2012-06-21

**Authors:** Zhihong Yang, Yuxia Zhang, Jongsook Kim Kemper, Li Wang

**Affiliations:** 1 Departments of Medicine and Oncological Sciences, Huntsman Cancer Institute, University of Utah School of Medicine, Salt Lake City, Utah, United States of America; 2 Department of Molecular and Integrative Physiology, University of Illinois at Urbana-Champaign, Urbana, Illinois, United States of America; German Cancer Research Center, Germany

## Abstract

We report here a novel interplay between tumor suppressor p53 and nuclear receptor SHP that controls p53 and SHP stability. Overexpression of p53 causes rapid SHP protein degradation, which does not require the presence of Mdm2 and is mediated by the proteosome pathway. Overexpressing SHP alone does not affect p53 stability. However, SHP destabilizes p53 by augmentation of Mdm2 ubiquitin ligase activity toward p53. The single amino acid substitution in the SHP protein SHPK170R increases SHP binding to p53 relative to SHP wild-type, whereas SHPG171A variant shows a diminished p53 binding. As a result of the cross-regulation, the tumor suppressor function of p53 and SHP in inhibition of colon cancer growth is compromised. Our findings reveal a unique scenario for a cross-inhibition between two tumor suppressors to keep their expression and function in check.

## Introduction

Small heterodimer partner (*SHP*, NROB2) plays a critical role in metabolic diseases [1], including bile acid biosynthesis [2], [3], bile acid and bile duct ligation (BDL) induced cholestatic liver injury [4], [5], fatty liver [6], [7], hypercholesterolemia [8], glucose metabolism [9], [10], obesity [11]–[13], and liver fibrosis [14]. The divergent roles of SHP are likely contributed by its regulation of various metabolic genes [15]–[18]. Our recent studies revealed another important role of SHP in regulation of miRNA expression and function [19]–[26]. In addition, growing evidence suggests SHP as a tumor suppressor [27] in hepatocellular carcinoma (HCC) by inhibiting hepatocyte proliferation [28], activating apoptosis [29], and repressing the expression of DNA methyltransferase [30], [31]. In spite of these important studies, the relationship between SHP and other tumor suppressors remains largely unexplored.

p53 tumor suppressor is a transcription factor that binds to its response elements in the *Mdm2* promoter to activate Mdm2 [32]. The p53 protein stability is reduced by Mdm2 via proteasomal degradation [33]. This creates an autoregulatory feedback loop between p53 and Mdm2. Recent studies show that post-translational modification of p53 and Mdm2, including phosphorylation [34], [35], is important for the function of p53 and Mdm2. Phosphorylation and acetylation of p53 disrupt its interaction with Mdm2, preventing p53 repression by Mdm2 [34], [36].

Our recent study identified Mdm2 as an activator of the ApoCIII promoter, which was repressed by p53 and SHP co-expression [37]. We further identified a cross-talk between SHP and Mdm2 via a feedback regulatory loop [38]. These studies raised an interesting question whether p53 and SHP cross-regulate each other's stability. In the present study, we provide the first evidence that p53 drastically decreases SHP protein stability independent of Mdm2, and SHP in turn destabilizes p53 through enhancing the activity of Mdm2. The findings provide new insight into the mechanisms of cross-regulation of protein stability between two tumor suppressors, which are important for our better understanding the tumor suppressor function of p53 and SHP.

## Results

### p53 causes rapid SHP protein degradation independent of Mdm2

**Figure 1:**
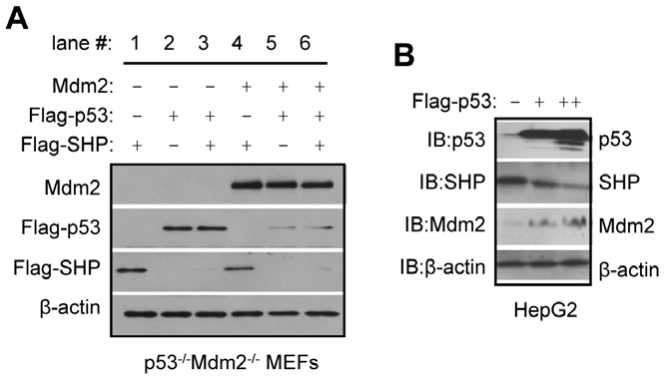
p53 causes rapid SHP protein degradation independent of Mdm2. A: Western blots to determine the effects of p53 and Mdm2 on SHP protein expression in *p53^−/−^Mdm2^−/−^* MEF cells lacking p53 and Mdm2. The *p53^−/−^Mdm2^−/−^* MEF cells were transfected with Flag-SHP, Flag-p53 or Mdm2 expression vectors, and Mdm2, p53 or SHP proteins were detected using anti-Mdm2 and anti-Flag antibodies, as indicated. The sizes of p53 and SHP proteins are distinct, thus both proteins were detected simultaneously using anti-Flag antibodies on the same blot. B: Western blots to determine the effect of p53 on endogenous SHP protein expression. Flag-p53 expression vectors were transfected in HepG2 cells, and p53 (both endogenous and exogenous), SHP (endogenous), and Mdm2 (endogenous) proteins were detected using anti-p53, anti-SHP and anti-Mdm2 antibodies, respectively.

We first determined whether p53 regulates SHP protein expression using *p53* and *Mdm2*-double-deficient *p53^−/−^Mdm2^−/−^* MEFs. This will exclude the potential influence of Mdm2. Co-expression of p53 and SHP markedly reduced SHP protein to an undetectable level (Fig. 1A, row 3, lane 3 *vs.* 2), suggesting that the downregulation of SHP by p53 does not depend on the presence of Mdm2. Similarly, ectopic expression of Mdm2 with SHP moderately decreased SHP (row 3, lane 4 *vs.* 1), suggesting that Mdm2 was able to downregulate SHP in the absence of p53. p53 protein was drastically decreased by Mdm2 (row 2, lane 5 *vs.* 2). However, SHP was degraded to the same extent in the presence of Mdm2 regardless of the level of p53 (row 3, lane 6 *vs.* 3), indicating an additive effect of Mdm2 and p53. Taken together, the results suggest that p53 and Mdm2 independently decrease SHP protein stability, likely by increasing its degradation.

We next determined whether p53 downregulates the endogenous SHP protein using HepG2 cells, because these cells express high basal levels of SHP [28]. As expected, overexpression of p53 reduced the levels of endogenous SHP protein in a dose-dependent fashion (Fig. 1B). In addition, the endogenous Mdm2 protein was upregulated by p53, consistent with p53's transactivation function.

**Figure 2:**
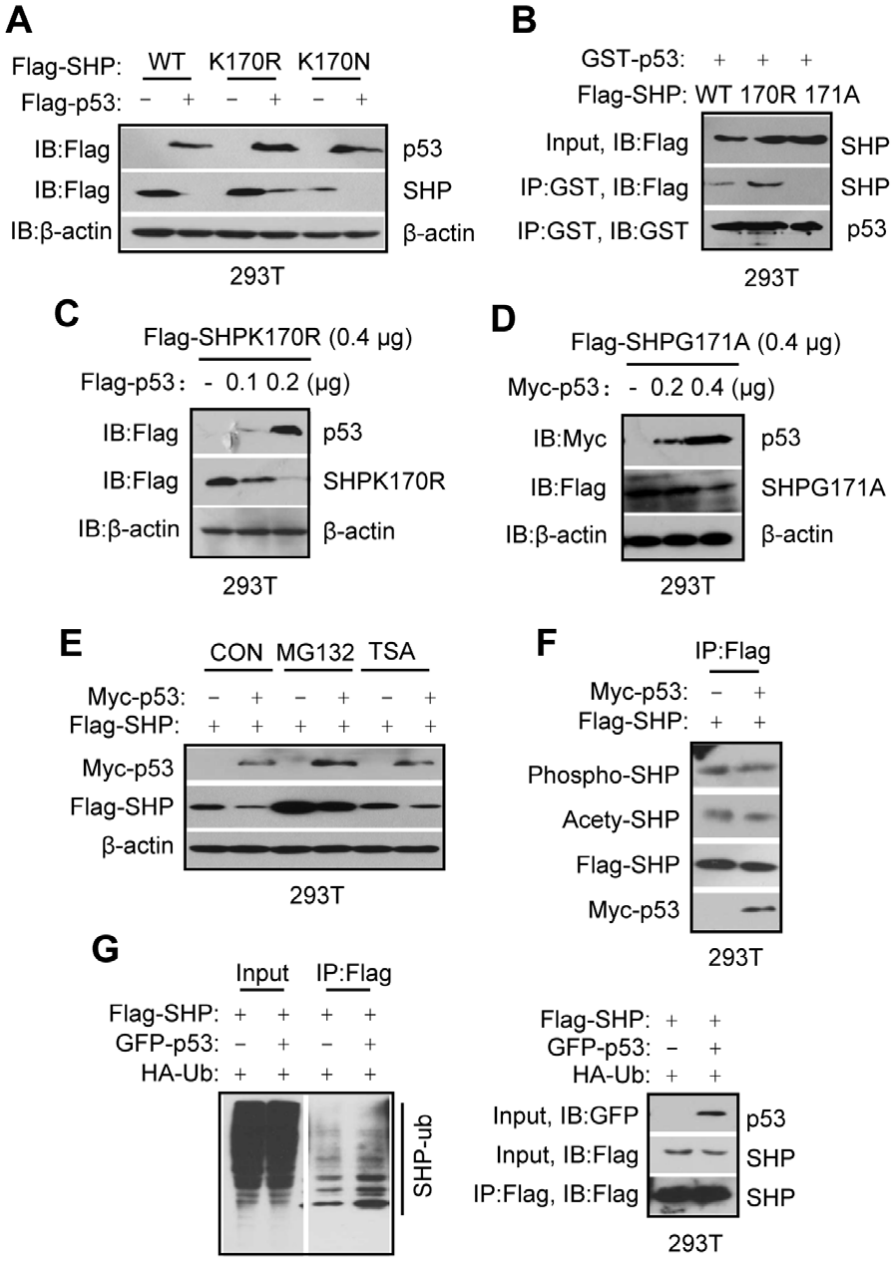
Downregulation of SHP protein by p53 is mediated by proteosome degradation. A: Western blots to determine the effect of p53 on SHPWT, SHPK170R, or SHPK170N protein expression. Flag-SHPWT, Flag-SHPK170R or Flag-SHPK170N expression vectors were transfected in 293T cells without or with Flag-p53 co-expression, and p53 and SHP proteins detected simultaneously using anti-Flag antibodies on the same blot. B: Immunoprecipitation and Western blots to determine the association of SHP proteins with p53 protein. Flag-SHPWT, Flag-SHPK170R or Flag-SHPG171A expression vectors were co-transfected with GST-p53 expression vector. Whole cell lysates were immunoprecipitated with the anti-GST antibodies, and SHP and p53 proteins were detected using anti-Flag or anti-GST antibodies, respectively. C: Western blots to determine the effect of p53 on SHPK170R protein levels. Flag-SHPK170R and Flag-p53 plasmids were cotransfected in 293T cells, and SHP and p53 proteins were detected using anti-Flag antibodies. p53 and SHP protein can be easily distinguished because of different molecular weight. D: Western blots to determine the effect of p53 on SHPG171A protein levels. Myc-p53 and Flag-SHPG171A plasmids were cotransfected in 293T cells, and p53 and SHPG171A proteins were detected using anti-Myc and anti-Flag antibodies, respectively. E–F: Western blots to determine the effect of p53 on SHP protein expression in the presence of MG132 or TSA (E) or on the phosphorylated and acetylated SHP protein expression (F). Flag-SHP expression vectors were transfected alone or together with Myc-p53 vectors, and both proteins were detected using antibodies as indicated in the figures. CON, control. G: *In vitro* Ubiquitination assays to determine the effect of p53 on SHP protein ubiquitination. Flag-SHP and HA-Ub expression vectors were transfected without or with GFP-p53 vectors. Whole cell lysates were immunoprecipitated with the anti-Flag antibodies, and ubiquitinated SHP was detected using anti-HA antibodies (indicated by a solid line).

### Downregulation of SHP by p53 is mediated by proteasome degradation

Recently we discovered several naturally occurring SHP mutations in humans [39], including SHPK170N and SHPG171A. We mutated K170N to K170R and tested their protein expression regulation by p53 in 293T cells. Interestingly, p53 reduced both SHPK170R and SHPK170N proteins (Fig. 2A), but decreased SHPK170R to a lesser extent than SHPWT.

Co-IP and Western blots revealed that SHPK170R showed increased binding to p53 relative to SHPWT, whereas the binding between SHPG171A and p53 was lost (Fig. 2B). The results suggest that the binding affinity of p53 to SHP is dramatically affected by a single amino acid substitution in the SHP protein.

We further confirmed that the SHPK170R protein was rapidly degraded by p53 in a dose-dependent fashion (Fig. 2C). In contrast, the amount of p53 plasmid (0.2 µg) that caused a complete SHPK170R degradation had no effect on SHPG171A levels and twice the amount of p53 (0.4 µg) caused only a partial reduction of SHPG171A (Fig. 2D). Thus, the ability of p53 to downregulate SHPG171A protein is severely impaired by the diminished interaction between SHPG171A and p53 in 293T cells.

The proteasome inhibitor MG132 markedly increased basal expression of SHP but did not completely block the ability of p53 to reduce SHP protein (Fig. 2E). The histone deacetylase inhibitor TSA, in contrast, did not affect SHP degradation by p53. In addition, neither the levels of phosphorylated nor acetylated SHP were altered by p53 (Fig. 2F). Furthermore, p53 increased the levels of ubiquitinated SHP protein (Fig. 2G). Collectively, the results suggest that p53 decreased SHP through the proteasome-mediated pathway.

### SHP does not interfere with p53 and Mdm2 interaction

**Figure 3:**
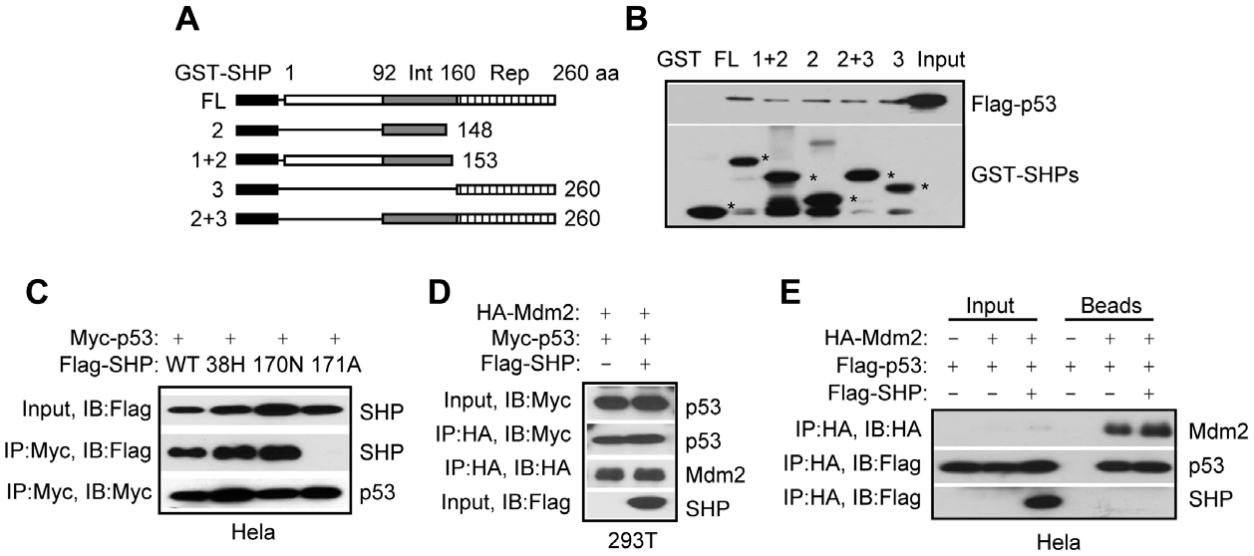
SHP does not interfere p53 and Mdm2 interaction. A–B: GST pull down to determine the *in vitro* interaction of SHP with p53. Flag-p53 was *in vitro* translated and used to interact with various GST-SHP fusion proteins, including GST-FL (full length), GST-2 (interaction domain), GST-1+2 (repression domain deletion), GST-3 (repression domain), and GST-2+3 (N-terminal domain deletion), that were expressed from bacterial Escherichia coli BL21/DE3/RIL. C: Immunoprecipitation and Western blots. Plasmids expressing Myc-p53 were cotransfected with Flag-SHPWT, Flag-SHP38H, Flag-SHP170N, and Flag-SHP171A plasmids in Hela cells. Co-IP and WB were performed with corresponding antibodies as indicated. D–E: Western blots to determine the effect of SHP on p53 and Mdm2 interaction. D: HA-Mdm2 (2 µg), Myc-p53 (4 µg) and Flag-SHP (2 µg) plasmids were cotransfected in 293T cells, and their proteins were detected using anti-HA, anti-Myc and anti-Flag antibodies, respectively. E: HA-Mdm2 (2 µg), Flag-p53 (4 µg) and Flag-SHP (2 µg) plasmids were cotransfected in Hela cells, and their proteins were detected using anti-HA and anti-Flag antibodies, respectively.

To further characterize the SHP and p53 interaction, we mapped the region of SHP required for the interaction using various GST-SHP deletion constructs (Fig. 3A). GST pull down assays revealed that the full length SHP (FL) and the repression domain of SHP (Rep) showed strongest binding to p53 (Fig. 3B). Interestingly, SHP N-terminal and its interaction domain (Int) also bound p53 with low affinity. As expected, the lost interaction of SHPK171A with p53 was also observed in Hela cells (Fig. 3C), whereas other SHP mutants (R38H and K170N) did not disrupt SHP and p53 interaction. The results are in agreement with our observation in 293T cells (Fig. 2B–2D), which supports our conclusion that specific amino acids rather than the entire domain of SHP may be more important for SHP and p53 interaction that dictates p53 functional regulation of SHP.

Due to the important relationship between p53 and Mdm2, we determined whether SHP affected the interaction between Mdm2 and p53 using Co-IP and Western blots. Overexpressing SHP failed to disrupt Mdm2 and p53 protein association in 293T (Fig. 3D) and Hela cells (Fig. 3E). Therefore, the binding affinity of SHP to p53 is weaker than the binding affinity of Mdm2 to p53.

### SHP destabilizes p53 protein by enhancing the activity of Mdm2

**Figure 4:**
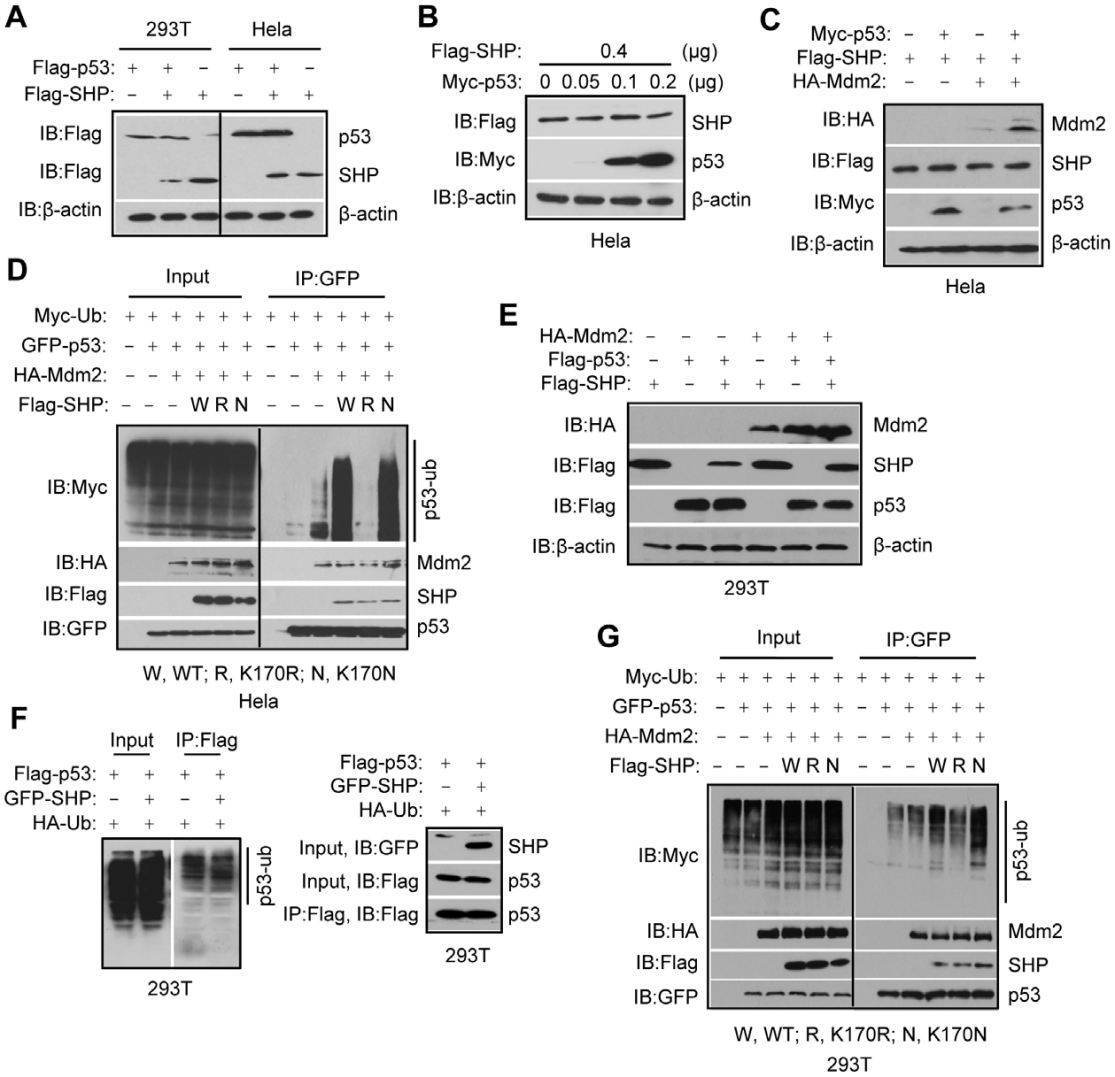
SHP destabilizes p53 protein by enhancing the activity of Mdm2. A: Western blots. Plasmids expressing Flag-p53 were co-transfected with Flag-SHP plasmids in 293T or Hela cells. p53 or SHP protein levels were determined using anti-Flag antibodies. B: Western blots. Flag-SHP and Myc-p53 plasmids were cotransfected in Hela cells, and SHP and p53 proteins were detected using anti-Flag and anti-Myc antibodies, respectively. C: Western blots. Myc-p53, Flag-SHP or HA-Mdm2 expression vectors were transfected in Hela cells, and Mdm2, SHP and p53 proteins were detected using anti-HA, anti-Flag or anti-Myc antibodies, respectively. D: Ubiquitination assays. Hela cells were cotransfected with GFP-p53, HA-Mdm2, and various Flag-SHP expression vectors together with Myc-ubiquitin (Myc-Ub) plasmids. GFP-p53 was immunoprecipitated from cell extracts with anti-GFP antibodies, and ubiquitinated p53 in the immunoprecipitates was detected by Western blots with anti-Myc antibodies. Positions of ubiquitinated p53 proteins are indicated by a solid line. E: Western blots. HA-Mdm2, Flag-p53 or Flag-SHP expression vectors were transfected in 293T cells, and Mdm2, SHP and p53 proteins were detected using anti-HA or anti-Flag antibodies, respectively. F: Ubiquitination assays. 293T cells were cotransfected with Flag-p53 and GFP-SHP expression vectors together with HA-ubiquitin (HA-Ub) plasmids. Flag-p53 was immunoprecipitated from cell extracts with anti-Flag antibodies, and ubiquitinated p53 in the immunoprecipitates was detected by Western blots with anti-HA antibodies. Positions of ubiquitinated p53 proteins are indicated by a solid line. G: Ubiquitination assays. 293T cells were cotransfected with GFP-p53, HA-Mdm2, and various Flag-SHP expression vectors together with Myc-ubiquitin (Myc-Ub) plasmids. GFP-p53 was immunoprecipitated from cell extracts with anti-GFP antibodies, and ubiquitinated p53 in the immunoprecipitates was detected by Western blots with anti-Myc antibodies. Positions of ubiquitinated p53 proteins are indicated by a solid line.

Thus far, we showed that p53 reduced SHP protein expression in several cell models, including *p53^−/−^Mdm2^−/−^* MEFs, HepG2 and 293T cells (Figs. 1, 2, 3). We also showed that the SHPG171A and p53 interaction was lost in 293T (Fig. 2B) and Hela cells (Fig. 3C), and that SHP did not interfere with p53 and Mdm2 interaction in both cells (Fig. 3D–3E). To determine whether SHP regulates p53 protein expression, we overexpressed both proteins in 293T and Hela cells. Interestingly, p53 did not modulate SHP protein levels in Hela cells (Fig. 4A), which was confirmed by a dose-dependent experiment (Fig. 4B). On the other hand, p53 protein was not altered by SHP co-expression in either cell types (Fig. 4A).

Based on the above results, Hela cells were used to examine whether SHP affected p53 protein in the presence of co-expressed Mdm2. To our surprise, p53 protein levels were decreased by SHP when Mdm2 was co-expressed (Fig. 4C, row 3, lane 4 *vs.* 2), which correlated with the increased Mdm2 levels (row 1, lane 4 *vs.* 3). We conducted *in vitro* ubiquitination assays to determine the effect of SHP on Mdm2 mediated p53 ubiquitination. As expected, p53 ubiquitination was significantly increased by SHP in the presence of Mdm2 (Fig. 4D). The effects of SHP variants on p53 ubiquitination were also compared. SHPK170R failed to increase p53 ubiquitination mediated by Mdm2. SHPK170N, on the other hand, further increased p53 ubiquitination compared with SHPWT.

**Figure 5:**
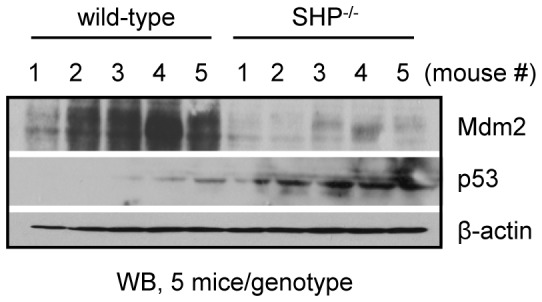
Mdm2 protein is upregulated and p53 protein is downregulated in *SHP^−/−^* mice. Western blots to determine endogenous p53 and Mdm2 proteins in the livers of wild-type and *SHP^−/−^* mice. Five mice per genotype were analyzed.

Because Hela cells express the HPV E6 protein, which may interfere with physiological p53 expression, we repeated the experiments in 293T cells. Similar to the results obtained in Fig. 4A, co-expression of SHP and p53 decreased SHP (Fig. 4E, row 2, lane 3 *vs.* 1), but not p53 protein (row 3, lane 3 *vs.* 2). As expected, the level of p53 was reduced by Mdm2 (row 3, lane 5 *vs.* 2). A consistently decreased p53 expression was observed in the presence of SHP and Mdm2 (row 3, lane 6 *vs.* 3), which likely affected p53's ability to downregulate SHP (row 2, lane 6 *vs.* 3). In vitro ubiquitination assays showed that SHP alone did not affect p53 uniquitination (Fig. 4F). However, Mdm2-mediated p53 uniquitination was enhanced when SHP was co-expressed (Fig. 4G). In addition, SHPK170R diminished whereas SHPK170N augmented p53 ubiquitination by Mdm2. SHP can be degraded by p53 in 293T but not Hela cells, which may contribute to the greater effect of SHP in Hela cells. Overall, the data demonstrate that SHP augments Mdm2-mediated p53 ubiquitination and degradation in both Hela and 293T cells.

### The protein expression of p53 and Mdm2 is altered in *SHP*-deficient mice

To provide physiological relevance of the cross-regulation between SHP and p53, we determined p53 and Mdm2 proteins in livers of wild-type and *SHP^−/−^* mice. Consistent with our prior observation that SHP enhances Mdm2 protein stability [38], the hepatic Mdm2 protein expression was markedly decreased by *SHP*-deficiency (Fig. 5). As expected, the levels of p53 protein were increased in *SHP^−/−^* livers compared with the wild-type mice. The results provide strong *in vivo* evidence for SHP induction of Mdm2 protein and reduction of p53 protein expression.

**Figure 6:**
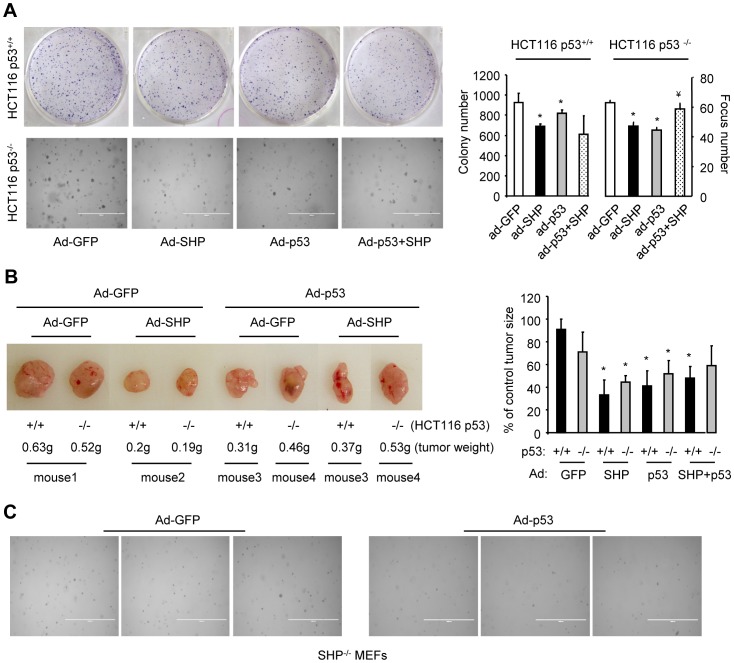
SHP and p53 inhibition of HCT116 tumor growth was compromised. A: Colony formation assays (upper panel) and foci formation assays (lower panel). Left: HCT116*p53^+/+^* and HCT116*p53^−/−^* cells were transduced with GFP, SHP or p53 adenoviruses (Ad) as indicated, and colony (upper) or foci (lower) formation was analyzed. The experiment was repeated 4 times (duplicate or triplicate/time) and one representative result is shown. Right: statistical analysis of the results on the left. Data are represented as mean ± SEM. *p<0.01 *vs.* white bar; ^¥^p<0.01 *vs.* black or grey bar. B: Tumorigenesis assays. HCT116*p53^+/+^* and HCT116*p53^−/−^* cells were transduced with GFP, SHP or p53 adenoviruses (Ad) and were either injected into the same or different mouse (left and right flanks). Each experimental group was injected into three flanks (one flank/mouse, three mice). Tumors were collected 2 weeks later. Left: representative tumor images from each group. Right: statistical analysis of tumor volume (n = 3/group). *p<0.01 *vs.* GFP group corresponding to *p53^+/+^* or *p53^−/−^* cells. C: Foci formation assays in *SHP^−/−^* MEF cells that were overexpressed with GFP control or p53 adenoviruses. Three representative images per group are shown.

**Figure 7:**
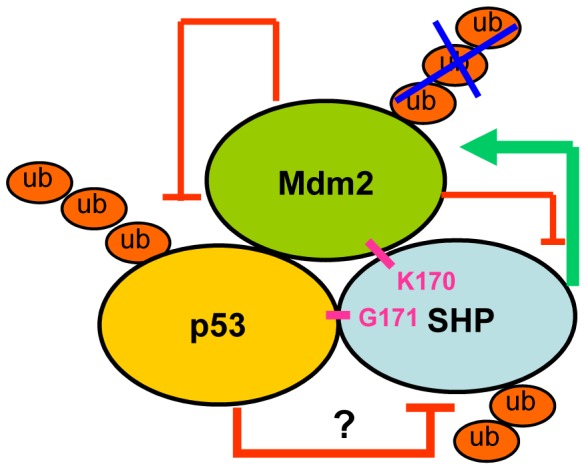
Diagram showing the cross-regulation among SHP, Mdm2 and p53. SHP interacts physically with Mdm2 through lysine 170 to markedly increase Mdm2 protein stability. The increase in Mdm2 destabilizes p53, which in part explains SHP augmentation of Mdm2-mediated p53 ubiquitination. In turn, p53 downregulates SHP by targeting the protein for proteasomal degradation through an undefined mechanism (indicated by a question mark). Mdm2 also moderately decreases SHP, and its effect is strongly augmented by *p53*-deficiency, thus creating an autoregulatory loop with SHP. Our study reveals a fine tuned interplay among SHP, Mdm2 and p53, which provides new insight into the mechanisms of regulating p53 and Mdd2 stability mediated by a nuclear receptor.

### SHP and p53 inhibition of HCT116 tumor growth is compromised

Like p53, our recent studies showed that SHP also functions as a tumor suppressor [27]–[29]. Because p53 and SHP destabilize each other, we examined the tumor suppressive effects of SHP and p53 on HCT116 tumor growth. Overexpression of either SHP or p53 decreased the number of colonies formed by HCT116*p53^+/+^* cells (Fig. 6A, upper panel). The individual effect of SHP and p53 was not further enhanced by co-expression of either protein. Foci formation assays showed that individual expression of SHP or p53 decreased foci numbers and that co-expression of both proteins reversed their individual effect in the HCT116*p53^−/−^* cells (Fig. 6A, lower panel). The *p53^+/+^* and *p53^−/−^* cells exhibited a similar ability to grow tumors in nude mice (Fig. 6B, tumor 2 *vs.* 1). Expression of SHP or p53 inhibited tumor growth in both *p53^+/+^* and *p53^−/−^* cells. The tumor weight tended to be higher in *p53^−/−^* cells co-expressing SHP and p53 than in cells expressing SHP alone. However, due to the variation of individual tumor volume, the difference did not reach significance. SHP degradation by p53 seems to be more dramatic when p53 is re-expressed in *p53*-deficient cells (*p53^−/−^* MEFs, Fig. 1A) than in cells that contain p53 (Fig. 2B, Fig. 3E), which may contribute to the differential outcome between *p53^+/+^* and *p53^−/−^* cells. In addition, overexpression of p53 markedly inhibited foci formation in *SHP*-deficient MEF cells (Fig. 6C).

## Discussion

In the present study, we provide convincing evidence for a cross-talk between p53 and nuclear receptor SHP. We show that p53 is able to downregulate SHP in MEFs, HepG2 and 293T cells, but not in Hela cells. On the contrary, SHP regulates p53 protein in a similar fashion in both 293T and Hela cells. SHP alone does not alter p53 protein expression, nor interfere with p53 and Mdm2 interaction, but it augments p53 degradation by Mdm2 through ubiquitination.

One prior report revealed a crosstalk among nuclear receptor TR3 (Nur77), p53, and Mdm2 [40]. Interestingly, TR3 binding to p53 blocks p53 ubiquitination mediated by Mdm2, while SHP downregulates p53 protein by increasing its degradation through Mdm2. Therefore, SHP and TR3 play distinct roles in regulating the p53-Mdm2 pathway.

We recently elucidated an autoregulatory feedback loop between Mdm2 and SHP proteins [38]. In the study, we showed that the endogenous SHP protein was co-immunoprecipitated with the endogenous Mdm2 and p53 protein in HepG2 cells using specific anti-SHP, anti-Mdm2 and anti-p53 antibodies. In the present study, we showed that the K170 and G171 residues in the SHP protein are important for SHP and p53 interaction to regulate p53-mediated SHP stability as well as SHP-mediated p53 stability via Mdm2. In addition, the Mdm2 protein was reduced whereas the p53 protein was induced in *SHP^−/−^* livers compared with the wild-type mice. Therefore, the SHP and p53 interaction has a clear physiological relevance.

As a consequence of the cross-inhibition between SHP and p53, an antagonizing effect between SHP and p53 was observed in HCT116*p53^+/+^* and *p53^−/−^* cells. The SHP and p53 in inhibiting tumor formation in both cells appear to be different, which is likely affected by the endogenous p53 function.

Our finding of decreased stability of SHP upon p53 overexpression is somewhat different from a recent report that stability of the SHP protein was increased in HepG2 cells treated with the p53 activator doxorubicin (DXR) [41]. However, the direct effect of p53 overexpression or knockdown on SHP stability was not tested. The discrepancy between the studies could be due to the different experimental conditions that were used.

Based on findings from our recently published study on SHP and Mdm2 crosstalk [38], and the present study on SHP and p53 crosstalk, we propose a fine tuned interplay among SHP, Mdm2 and p53 (Fig. 7). SHP interacts physically with Mdm2 through lysine 170 to markedly increase Mdm2 protein stability. The increase in Mdm2 destabilizes p53, contributing to SHP augmentation of Mdm2-mediated p53 ubiquitination and degradation. In turn, p53 downregulates SHP by targeting the protein for proteasomal degradation, through an undefined mechanism. Mdm2 also moderately decreases SHP, and its effect is strongly augmented by *p53*-deficiency, thus creating an autoregulatory loop with SHP. It should be noted that the interaction between p53 and Mdm2 is much stronger than the interaction of SHP with p53 or Mdm2. Presumably then the reciprocal regulation of p53 and Mdm2 predominates over the interaction with SHP when all three proteins co-exist. Our study provides new insight into the mechanisms controlling p53 and Mdm2 stability mediated by a nuclear receptor.

Overall our results suggest that depending on the cellular context and relative levels of the three proteins, SHP, p53, and Mdm2 may act in concert to determine susceptibility to carcinogenesis.

## Materials and Methods

### Plasmids, cell lines and reagents

Plasmids and cells were obtained from Drs. Takeshi Imamura (HA-Ub) [42], Yang Shi (Myc-Ub) [43], Paul Neilsen (Myc-p53) [44], Guillermina Lozano (*p53^−/−^Mdm2^−/−^* MEFs) [45], and David Jones (HCT116*p53^+/+^*, and HCT116*p53^−/−^* cells). Other plasmids or cell lines were from our laboratory [39] or purchased from Addgene. Antibodies against human MDM2, HA-tag, and β-actin were purchased from Sigma. p53 antibody was obtained from Santa Cruz. M2 antibody, anti-Flag M2 Magnetic Beads and anti-HA agarose were purchased from Sigma. Mouse monoclonal antibody against Myc-tag was purchased from Cell Signaling. MG132 was purchased from Cayman.

### Cell culture and transfection

*p53^−/−^Mdm2^−/−^* MEFs, HepG2, HEK293T, Hela, HCT116*p53^+/+^*, and HCT116*p53^−/−^* cells were cultured in Dulbecco's modified Eagle's medium (DMEM) with 10% fetal bovine serum. Transfection was performed by Lipofectamine 2000 according to the manufacturer's instructions. Colony formation assay [22], [28] was performed as described in our previous publications.

### Co-immunoprecipitation and Western blots

HEK293T cells were transfected with the indicated plasmids using Lipofectamine 2000 (Invitrogen), lysed in 500 µl lysis buffer and immunoprecipitated with anti-Flag M2 Magnetic Beads (Sigma) or anti-GFP antibodies for 4 hr at 4°C. The beads were washed four times with the lysis buffer. The bound proteins were separated by SDS-PAGE, followed by Western blotting with the indicated antibodies according to the standard procedures.

### Ubiquitination assay

Flag-SHP, GFP-p53, HA-Mdm2, or GFP-SHP, Flag-p53, HA-Mdm2, as indicated in each figure, were transfected with HA-ubi or myc-ubi into HEK293T cells for 24 hr, then treated with 5 mM MG132 (Cayman) for additional 6 hr. Cells were harvested and lysed in 500 ul of lysis buffer and immunoprecipitated with anti-Flag M2 magnetic beads (Sigma) or anti-GFP antibodies for 4 hr at 4°C. The beads were washed three times with the lysis buffer and analyzed with anti-GFP, anti-Flag, anti-HA, or anti-myc antibodies by Western blots, as indicated in the figures.

### Foci Formation

HCT116 cells (1×10^6^) were infected with adenoviruses as indicated in the figure legends for 2 hr (multiplicity of infection, 100). Cells grew for 1 day and 2.5×10^4^ cells were suspended with 1.5 ml of 0.4% top agar and 2×McCoy's 5α medium before being poured onto 6 well tissue culture plate coated with 1.5 ml of 0.7% bottom agar. The plates were prepared in triplicate. Ten days later, 6 areas per plate were chosen randomly and the number of visible colonies was counted and used for statistical analysis.

### Mouse Xenograft Model

Six-week-old female athymic nude mice nu/nu were used for HCT116 tumor xenografts. Both flanks of each mouse were injected with 1×10^6^ cells mixed with Matrigel Matrix HC (BD biosciences) in a total volume of 200 µl. After 2 weeks, tumors were dissected out and weighed. Protocols for animal use were approved by the Institutional Animal Care and use Committee at the University of Utah (IACUC number: 10-06010, approved 06-13-2011).

### Statistical analysis

Data are expressed as mean ± SEM. Statistical analyses were carried out using Student's unpaired t test; p < 0.01 was considered statistically significant.
